# Issues with RNF43 antibodies to reliably detect intracellular location

**DOI:** 10.1371/journal.pone.0283894

**Published:** 2023-04-06

**Authors:** Shanshan Li, Ruyi Zhang, Marla Lavrijsen, Thierry P. P. van den Bosch, Maikel P. Peppelenbosch, Ron Smits

**Affiliations:** 1 Department of Gastroenterology and Hepatology, Erasmus MC Cancer Institute, University Medical Center Rotterdam, Rotterdam, The Netherlands; 2 Department of Pathology, Erasmus MC Cancer Institute, University Medical Center Rotterdam, Rotterdam, The Netherlands; University of South Florida, UNITED STATES

## Abstract

RNF43 is an important negative regulator of β-catenin signaling by removing Wnt-receptors from the membrane. It is often mutated in cancers, leading to aberrant Wnt-dependent nuclear translocation of β-catenin. RNF43 has also been suggested to regulate β-catenin signaling directly within the nucleus, among other proposed nuclear functions. Given the importance of RNF43 in regulating Wnt/β-catenin signaling and its potential therapeutic relevance, a proper understanding of RNF43 biology is required. However, the presumed nuclear location is mainly based on available antibodies. These same antibodies have also been used extensively for immunoblotting or immunohistochemical purposes. However, a proper evaluation of their quality to reliably detect endogenous RNF43 has not been performed. Here, using genome editing we have generated a cell line that entirely misses *RNF43* exons 8 and 9, encoding the epitopes of commonly used RNF43 antibodies. Using this clone in addition to various other cell line tools, we show that four RNF43 antibodies only yield non-specific signals when applied in immunoblotting, immunofluorescence and immunohistochemical experiments. In other words, they cannot reliably detect endogenous RNF43. Our results suggest that the nuclear staining patterns are an antibody artifact and that RNF43 is unlikely to localize within the nucleus. More generally, reports using RNF43 antibodies should be interpreted with caution, at least for the RNF43 protein aspects described in these papers.

## Introduction

Aberrant activation of Wnt/β-catenin signaling is observed in a large number of cancers originating from various tissue types [1]. In normal cells the RNF43 protein, and its homologue ZNRF3, play an important role as negative regulators of this pathway. They encode for transmembrane E3-ubiquitin ligases that remove Wnt receptors from the membrane, thereby limiting the nuclear signaling of β-catenin [2, 3]. RNF43 mutations affecting protein function have been observed in a variety of cancers among which those of the colon, stomach, pancreas, endometrium and ovarium [2]. The functional consequence of these mutations is considered to be a reduced capability to remove Wnt-receptors, making such cancers hypersensitive to Wnt-ligand activation. For that reason these RNF43-mutant cancers have gained substantial therapeutic interest, as they may identify tumors that respond to extracellular Wnt-inhibitors that have been developed, such as FZD antibodies and Wnt-secretion Porcupine inhibitors [4].

Besides this well-established role of RNF43, it has also been linked to various other cellular functions. Loregger et al. suggested that RNF43 tethers TCF7L2 (also known as TCF4) to the inside of the nuclear membrane, thereby limiting β-catenin signaling directly inside the nucleus [5]. Other demonstrated nuclear functions are a role in the DNA damage response by potentially ubiquitinating γH2AX [6], a well-known marker of DNA damage, and suppressing TP53-dependent induction of P21 and BAX [7]. A direct binding of RNF43 to these nuclear proteins can only be shown by strong RNF43 overexpression. However, in support of these findings, nuclear staining patterns are observed using the commercially available HPA008079 and “home-made” 8D6 RNF43 antibodies [5, 6]. These findings may have important therapeutic implications as it may indicate that RNF43 mutations contribute to tumor growth through other mechanisms than solely affecting the level of Wnt receptors.

These and other antibodies have also been instrumental in immunohistochemical approaches to evaluate RNF43 protein expression in various tumor types. For example, using such antibodies RNF43 protein was observed to be over-expressed in liver cancers [8], lost in a subset of gastric and colorectal tumors [9, 10], and to correlate with gastric and clear cell renal cancer patient survival [11–13]. They have also been used to evaluate its expression in pancreatic lesions in relation to *RNF43* mutation status [14–16]. In basically all these examples nuclear staining patterns were observed, thus supporting a nuclear location of RNF43. These antibodies have also been used extensively in various reports to detect endogenous RNF43 through immunoblotting or immunofluorescence.

Given the importance of RNF43 in regulating Wnt/β-catenin signaling and its potential therapeutic relevance, a proper understanding of RNF43 biology is required. Antibodies are important instrumental tools for this purpose by detecting changes in RNF43 protein levels or intracellular location in various cell lines or tissue samples. However, a proper evaluation of their quality to reliably detect endogenous RNF43 has not been performed. Here, using genome editing we have generated a cell line that entirely misses *RNF43* exons 8 and 9, encoding the epitopes of three commonly used RNF43 antibodies. Using this clone in addition to various other cell line tools, we show that these and one additional RNF43 antibody recognizing a N-terminal epitope only yield non-specific signals when applied in immunoblotting, immunofluorescence and immunohistochemical experiments. Our results suggest that the nuclear staining patterns are an antibody artifact. In addition, reports using RNF43 antibodies should be interpreted with caution, at least for the RNF43 protein aspects described in these papers.

## Results

### Cell line tools to evaluate quality of RNF43 antibodies

**Fig 1:**
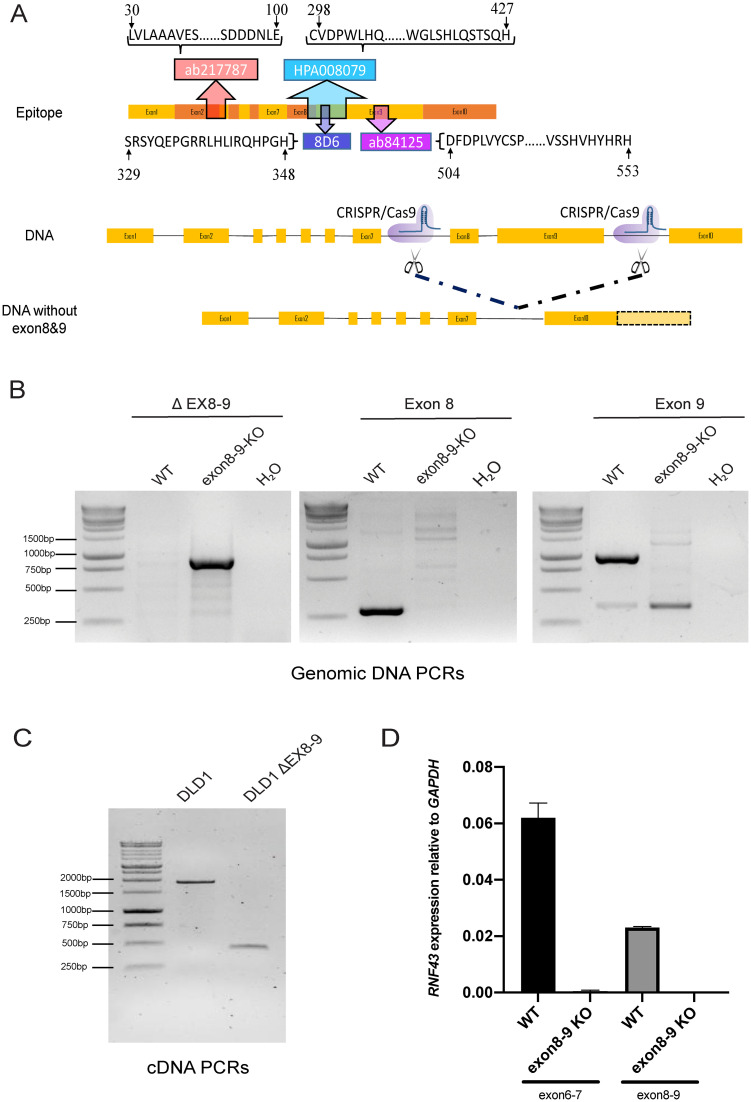
Epitope location and generation of DLD-1 RNF43 ΔEX8-9 clone. A. Schematic representation of *RNF43* mRNA and epitope location of RNF43 antibodies. The ab217787 antibody was raised against a N-terminal epitope encoded by exons 2 and 3, while the other three antibodies were raised against epitopes encoded by exons 8 and 9. A DLD-1 cell clone was generated that entirely misses these exons leading to a p.(Glu284_Pro769delext*56) deletion on protein level. B. Confirmation of correct deletion of exons 8 and 9 on DNA level. Left panel shows PCR with primers flanking the deletion. The expected approximate 900bp fragment is observed in the ΔEX8-9 clone, while the original 4kb fragment is too big to be amplified. Middle and right panels show, respectively, PCRs for exons 8 and 9, leading to the expected 283 and 914bp fragments in the wild-type cell line, whereas only non-specific bands are observed in the ΔEX8-9 clone. DNA marker used is the 1kb DNA ladder from Promega (#G5711) C. Confirmation of correct deletion of exons 8 and 9 on mRNA level. Primers flanking exons 8 and 9 reveal the expected 1904 and 445bp fragments, respectively, for the wild-type cells and ΔEX8-9 clone. D. A quantitative RT-PCR analysis of *RNF43* exons 8–9 shows undetectable levels in the ΔEX8-9 clone. Interestingly, as shown by a qRT-PCR for exons 6–7, total *RNF43* levels are decreased about 200-fold in this clone. In conclusion, we have successfully generated a DLD-1 clone that shows strongly reduced levels of *RNF43* mRNA entirely lacking exons 8 and 9.

Four commonly used RNF43 antibodies (rabbit polyclonal ab217787, rabbit polyclonal ab84125, rabbit polyclonal HPA008079 and rat monoclonal 8D6) were evaluated for their reliability to correctly detect RNF43. Their epitope locations are plotted in Fig 1A, showing that ab217787 maps to residues encoded by exons 2 and 3, and the other three antibodies all map to residues encoded by exons 8 and 9. For antibody testing we established a panel of cell lines and clones thereof. As positive controls for full-length RNF43 we used HT-29, and transient RNF43 transfection of HEK293T cells. DLD-1 and KM12 cells are, respectively, heterozygous and homozygous for a p.G659fs*41 mutation, which is expected to lead to a truncated protein that can be detected by all four antibodies theoretically. As negative controls we used HCT116 cells carrying a homozygous truncation at p.R117fs*41, and RNF43-KO KM12 cells [17]. Lastly, to fully exclude that RNF43 epitopes are generated from currently unknown promoters or alternative usage of internal ATG start sites, we generated a DLD-1 cell clone completely lacking exons 8 and 9 (deletion of amino acids 284–769). PCRs on genomic DNA confirmed the absence of both exons (Fig 1B), while on RNA level the expected shortened cDNA fragment was observed (Fig 1C). Sequencing of the shortened genomic and cDNA PCR products revealed the expected loss of exons 8 and 9. A quantitative RT-PCR analysis validated the absence of exons 8 and 9 (Fig 1D). Interestingly, a qPCR for exons 6 and 7 retained in the transcript, showed that overall *RNF43* RNA levels are decreased about 200-fold. In conclusion, we have successfully generated a DLD-1 clone that shows strongly reduced overall levels of *RNF43* mRNA entirely lacking exons 8 and 9.

### Non-specific binding of RNF43 antibodies revealed by immunoblotting

**Fig 2:**
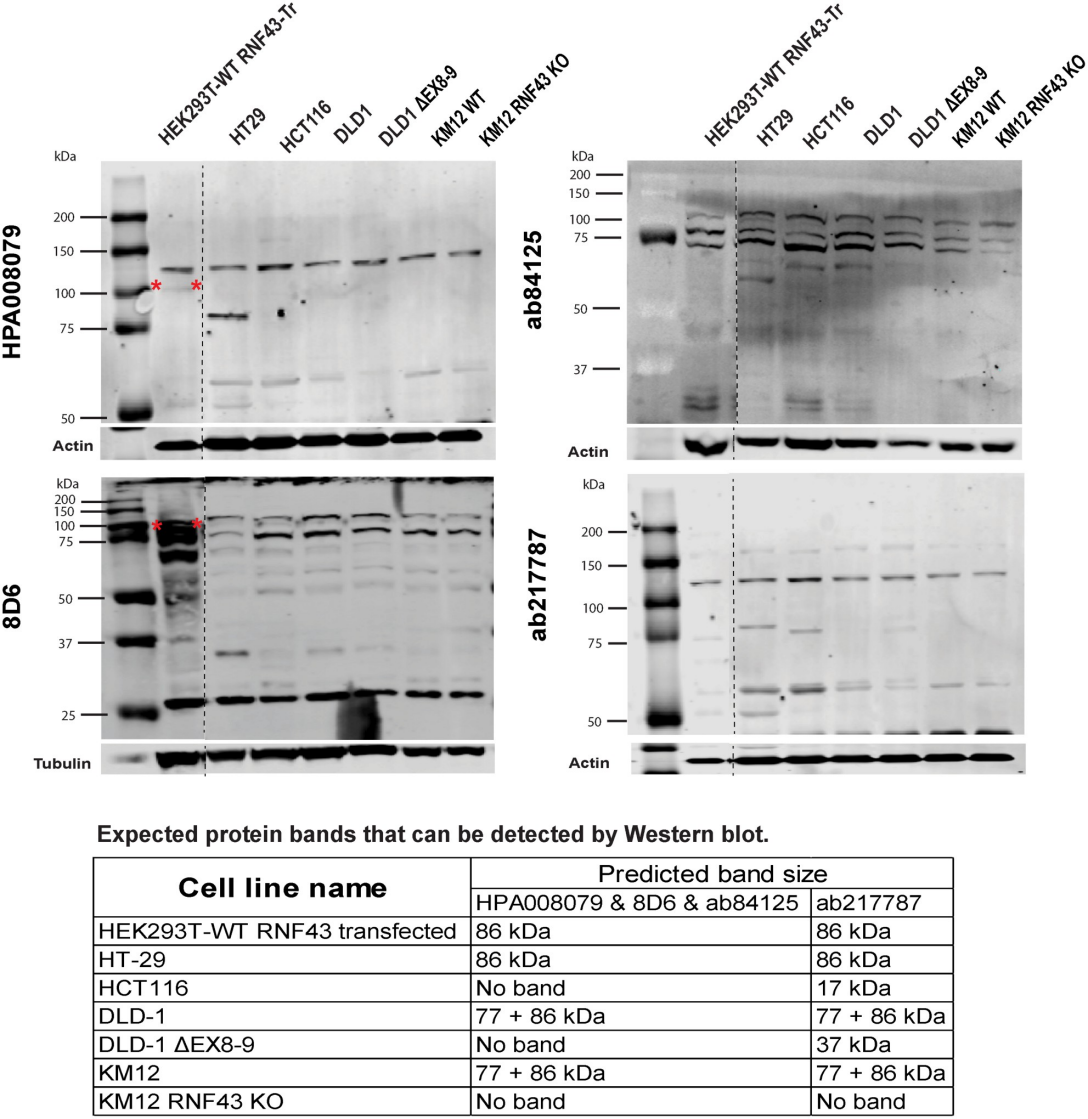
Immunoblot analysis of RNF43 antibodies. The HPA008079, ab84125, 8D6 and ab217787 antibodies cannot specifically recognize endogenous RNF43 by immunoblotting. No signal is expected in the HCT116, DLD-1 ΔEX8-9, and KM12 RNF43 KO lanes for the exon 8–9 located antibodies, while ab217787 may detected 17 and 37 kDa truncated bands in, respectively, HCT116 and DLD-1 ΔEX8-9. All antibodies may detect a specific truncated band in the DLD-1 and KM12 WT lanes. However, only non-specific bands are observed. The 8D6 and HPA008079 antibodies are able to detect overexpressed RNF43 (lanes 1). The dashed lines demarcate a non-essential protein marker and/or sample lane that were removed from the image. The table at the bottom shows the expected protein bands that can be detected for each sample. Protein sizes are based on transcript ID ENST00000407977.7 and protein ID CCDS11607.1. Overexpressed RNF43 is marked with a red asterisk. Original images can be found in S1 Fig.

Protein lysates from the cell lines described above, were used for immunoblotting (Fig 2 and S1 Fig). In all cases bands were identified that could be mistaken for RNF43. However, these same bands were also observed in the three negative control lines in our panel (HCT116, DLD-1 ΔEX8-9, and RNF43-KO KM12), clearly showing that the bands identified are not RNF43. Overexpressed RNF43 can be detected by the HPA008079 and 8D6 antibodies, while this was not the case for ab84125 and ab217787. Taken together, we conclude that these four antibodies are not suitable to identify RNF43 expressed at endogenous levels using immunoblotting.

### Evaluation of RNF43 antibodies for immunofluorescence approaches

**Fig 3:**
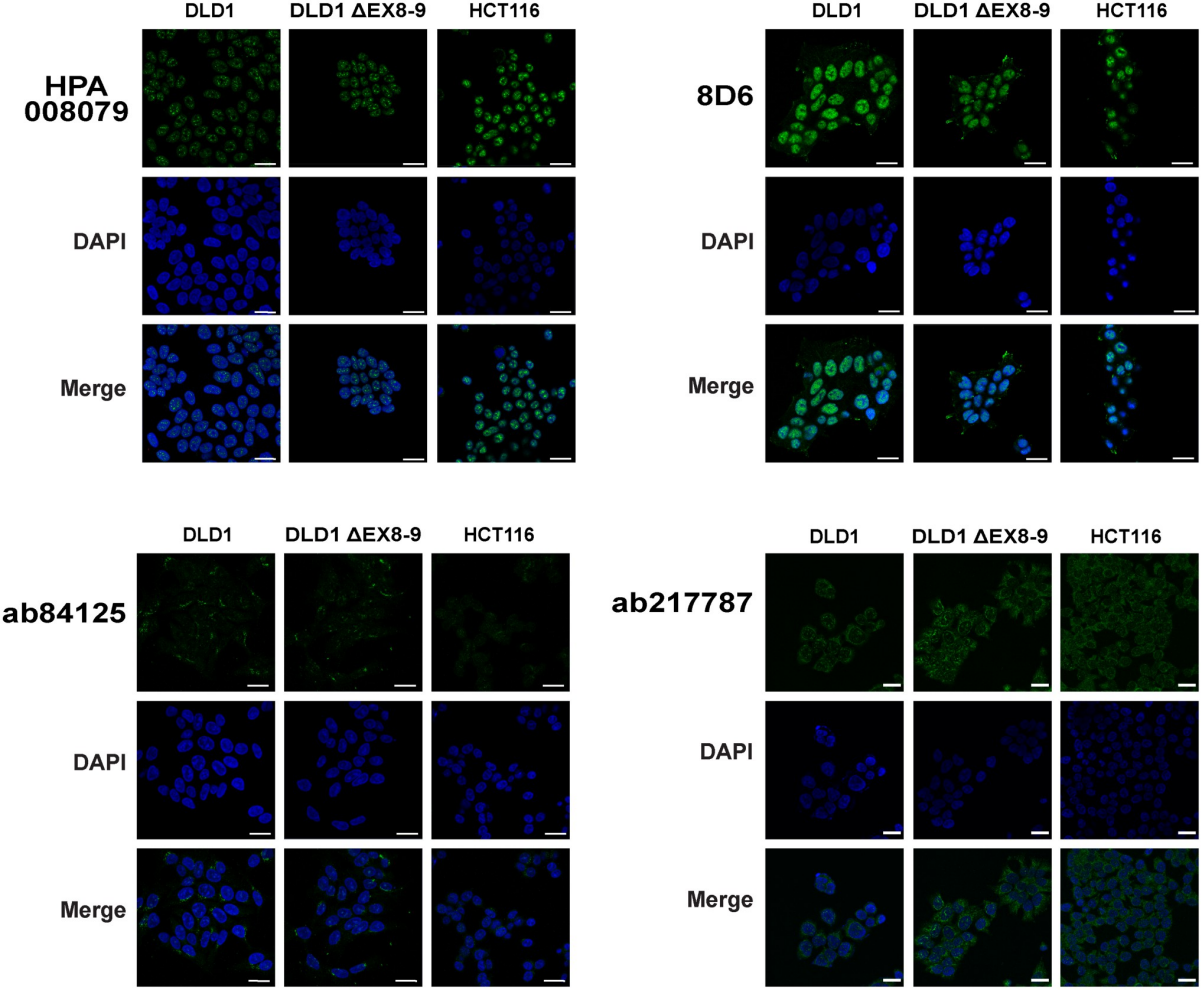
Immunofluorescence analysis of RNF43 antibodies. DLD-1, DLD-1 ΔEX8-9, and HCT116 cells were cultured on glass slides and stained with HPA008079, ab84125, ab217787and 8D6 antibodies. DLD-1 ΔEX8-9 and HCT116 cells are not expected to reveal any staining, but in all cases signals are observed comparable with the wild-type control DLD1. DAPI was used to stain the nuclei. For the 8D6 antibody the DAPI-staining is coincidentally stronger in the DLD-1 ΔEX8-9 cells, giving the potential false impression that the green 8D6 signal is weaker in these cells compared with their wild-type controls. However, evaluation of multiple independent images shows that non-specific signals are of comparable intensity. Larger images, a higher intensity image for ab84125, and negative control test are shown in S2 Fig. Scale bar, 25um.

RNF43 immunofluorescence (IF) has been used to demonstrate a nuclear, endoplasmic reticulum (ER) or membranous location of RNF43. We used DLD-1, DLD-1 ΔEX8-9, and HCT116 cells to validate these observations. The HPA008079 antibody showed a prominent non-specific nuclear staining in all cell lines, including both negative controls (Fig 3 and S2 Fig). Similar results were obtained for 8D6, which in addition showed a non-specific staining of cellular protrusions. The ab84125 antibody revealed a weaker non-specific punctate staining of a structure adjacent to the nucleus, reminiscent of the Golgi or endoplasmic reticulum. Ab217787 detected a non-specific nuclear structure and diffuse cytoplasmic staining. In theory this antibody may detect the 17 kDa truncated protein predicted to be present in HCT116 cells. However, given that it cannot recognize even overexpressed RNF43 by immunoblotting, the observed signal is highly likely to be non-specific. As for the DLD-1 ΔEX8-9 clone, also in this case it may detect the predicted 37 kDa truncated band. This clone shows however a more than 200-fold reduced expression of *RNF43*, meaning that the expected signal intensity should be strongly reduced. As this is not the case, we conclude that for this and all three other antibodies, only non-specific IF signals can be obtained.

**Fig 4:**
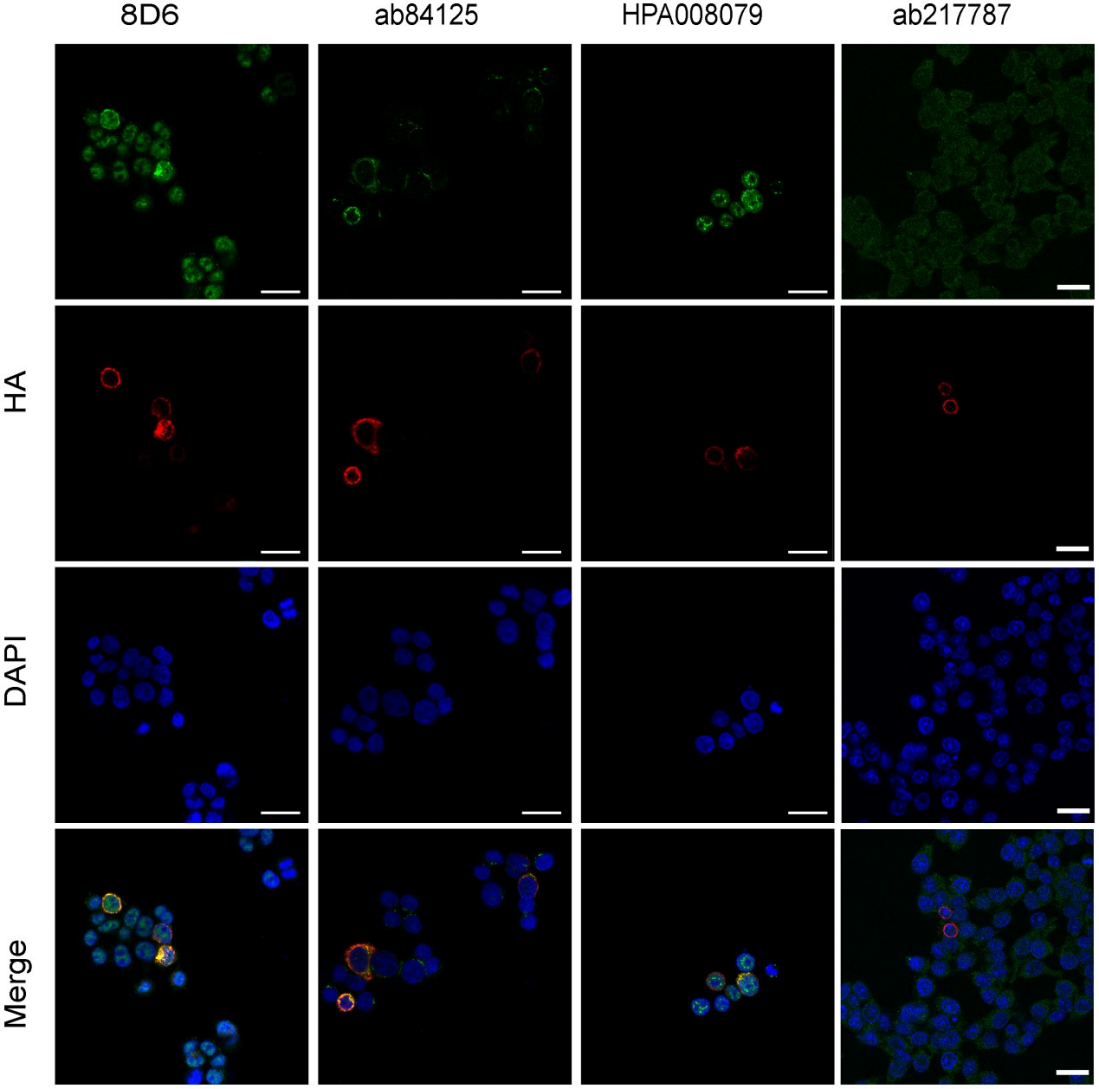
RNF43 antibodies can detect overexpressed RNF43 using immunofluorescence. HCT116 cells were transiently transfected with HA-tagged RNF43 and simultaneously stained with each RNF43 antibody and a HA-tag antibody. DAPI was used to stain the nuclei. Overexpressed RNF43 was present in the cytoplasm and detectable with all antibodies to some extent, except for the ab217787 antibody. Scale bar, 25um.

Next, we tested if overexpressed RNF43 can be detected using these antibodies. To this aim, HCT116 cells were transiently transfected with HA-tagged RNF43. Staining for the HA-tag showed a clear cytoplasmic pattern (Fig 4), in line with an ER-location reported previously for overexpressed RNF43 [17, 18]. Overexpressed RNF43 was also detected by the HPA008079, ab84125 and 8D6 antibodies, while ab217787 was unable to detect overexpressed RNF43. Taken together, this shows that three antibodies have some potential to identify RNF43 using IF, but at endogenous levels they fail to do so in a specific manner. We also wish to stress that this latter overexpression experiment is not intended to reveal the correct endogenous RNF43 location.

### Immunohistochemical staining with RNF43 antibodies

**Fig 5:**
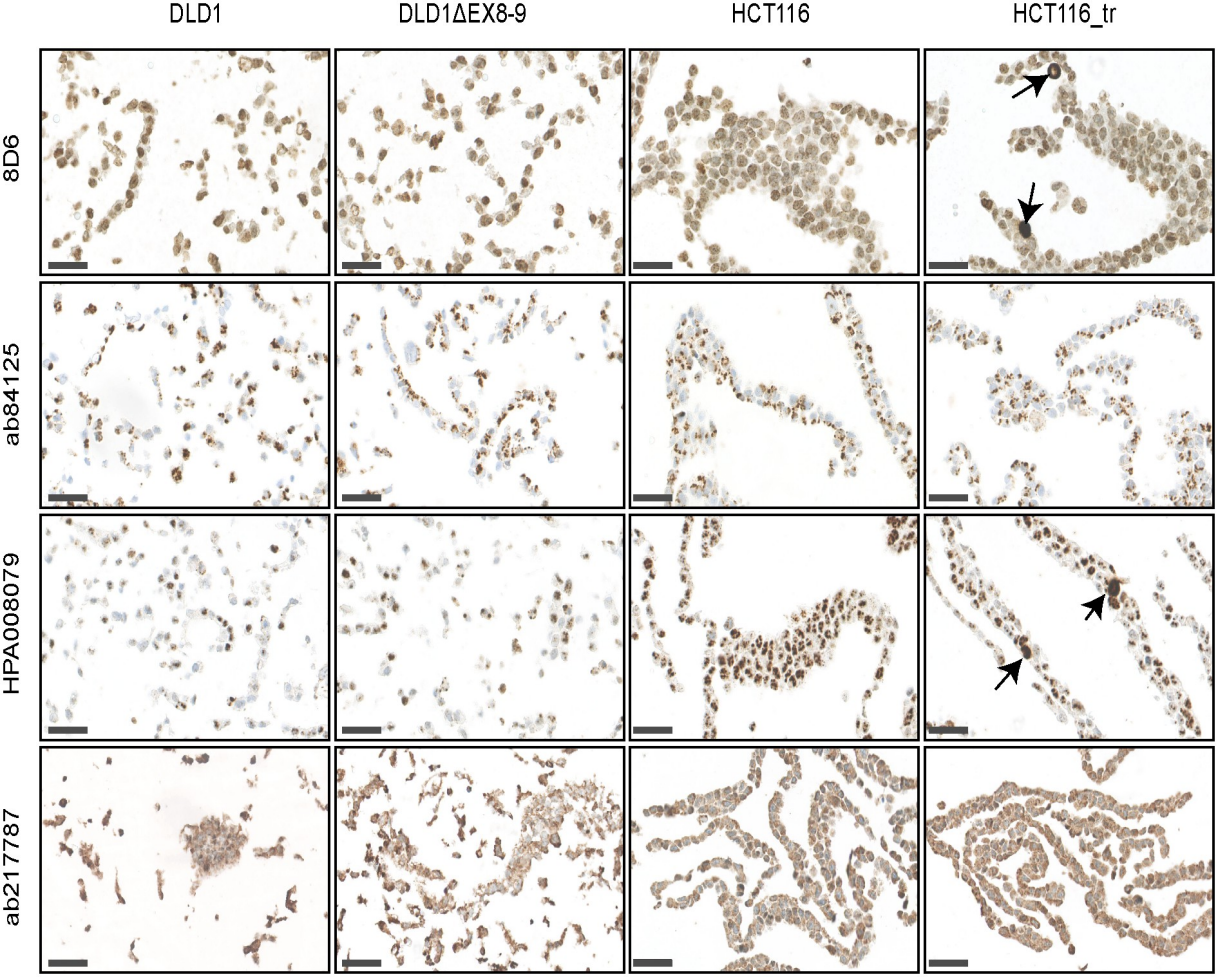
Immunohistochemical analysis of RNF43 antibodies. The indicated cell lines were formalin-fixed and embedded in paraffin. HCT116 cells transiently overexpressing RNF43 were also included. All antibodies give the same non-specific staining pattern as observed in the IF-experiments, showing that they all strongly recognize a non-specific protein not being RNF43. The 8D6 and HPA008079 antibodies can detect overexpressed RNF43, indicated by arrows, while this is not the case for ab84125 and ab217787. Scale bar, 50um.

The HPA008079, ab84125 and ab217787 antibodies have been commonly used on formalin-fixed and paraffin embedded (FFPE) tissue sections, among others to show loss or retainment of RNF43 protein expression in tumor samples. We generated FFPE tissue blocks from the same cell lines used for IF. As shown in Fig 5, basically identical non-specific staining patterns were observed as seen in the IF-experiments. Again HPA008079 and 8D6 showed a prominent non-specific nuclear staining and were able to detect overexpressed RNF43, while ab84125 and ab217787 non-specifically recognized similar structures as observed in IF. Thus, also the IHC analysis shows that all four antibodies recognize strongly other proteins not being RNF43.

### Generation and analysis of RNF43-3xFLAG knockin cell lines

**Fig 6:**
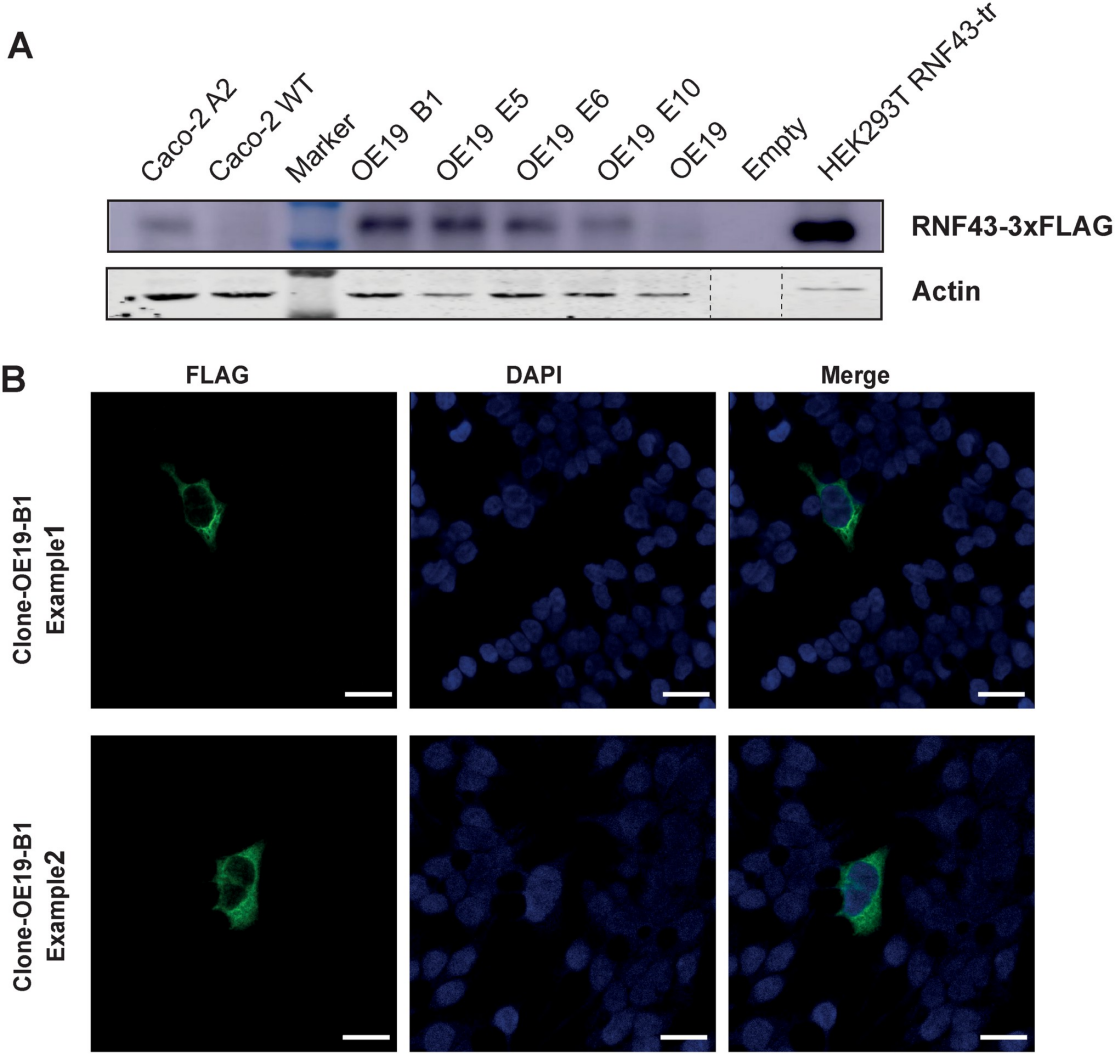
Immunoblot analysis and IF of 3xFLAG RNF43 knockin clones. A. Immunoblot analysis of Caco-2 and OE19 clones with a 3xFLAG knockin at the C-terminus of RNF43. A sensitive ECL Ultra Western HRP Substrate system was required to reveal the bands. A 30x diluted sample of RNF43-FLAG transfected HEK293T cells is added as control. B. Using a Tyramide SuperBoost system a small number of 3xFLAG positive OE19 cells could be identified. No positive cells were observed in the parental OE19 cell line. The staining pattern resembles that of overexpressed RNF43 showing a cytoplasmic and perinuclear pattern, the latter reminiscent of an ER location. However, given the low number of positive cells in only one cell line, we cannot draw a reliable generalized conclusion about RNF43’s intracellular location. Scale bar, 25um.

Thus far our experiments show that commonly available RNF43 antibodies cannot faithfully determine the intracellular location of RNF43. This means that our current knowledge depends on overexpression experiments that are more prone to artifacts. Therefore, to obtain reliable information on RNF43’s intracellular location, we generated OE19 and Caco-2 cells with knockin of a 3xFLAG, which is one of the most sensitive tags available with highly specific antibodies. OE19 and Caco-2 cells were chosen because they are among the highest *RNF43* expressing cell lines (https://www.proteinatlas.org/ENSG00000108375-RNF43/cell+line), which was confirmed using qPCR. Based on DNA and cDNA analysis, we successfully inserted the 3xFLAG tag preceded by a linker at the C-terminus of RNF43. However, immunoblotting using the fluorescent Odyssey system failed to detect endogenous FLAG-tagged RNF43 in both cell lines. This could be accomplished using a highly sensitive ECL Ultra Western HRP Substrate system (Fig 6A), nevertheless indicating that RNF43 protein expression is likely to be extremely low.

### Immunofluorescence analysis of endogenously tagged RNF43 cells

Next, we aimed at determining RNF43-3xFLAG’s intracellular location using IF. However, using conventional secondary antibodies we failed to obtain specific signals, despite testing various fixation methods. As a last resort, we used a tyramide signal amplification method. Caco-2 RNF43-3xFLAG cells remained negative using this method, while for OE19 we identified sparse cells showing a diffuse cytoplasmic staining specifically in 3xFLAG knockin clones (Fig 6B). It should however be noted that at most a handful positive cells were identified among all the cells present in a chamber of a Lab-Tek II chamber slide. Taken together, these results suggest that it is extremely difficult to faithfully detect endogenous RNF43 using IF and other methods. When detected, its location appears to be cytoplasmic, while no clear staining is observed in the nucleus.

## Discussion

Antibodies can be valuable tools for research. However, despite decades of warnings that the quality of antibodies should be carefully validated, it appears that more than half of the antibodies on the market are not specific to their target [19, 20]. Merely showing a band of the approximate expected size on a cropped western blot, is no guarantee that the correct target protein is identified.

Unfortunately, the same appears to be true for RNF43, an important negative regulator of Wnt signaling. We have evaluated four antibodies for their ability to reliably detect RNF43 protein. However, our results show that none of these antibodies is able to correctly detect endogenous RNF43 when applied in immunoblotting, IF and IHC experiments. Using these three methods on cell lines that express various lengths of RNF43 or that entirely miss the exons encoding the RNF43 epitopes, reveals in all cases exclusively non-specific signals for endogenous RNF43. Which non-specific proteins are recognized is currently unknown.

This has important implications, especially for its supposed nuclear location, an observation based largely on these antibodies. We consider a nuclear location unlikely for the following reasons. First, all overexpression studies yield cytoplasmic staining patterns, and show extensive overlap with markers of the ER [5, 7, 17, 18, 21, 22]. Some research groups have interpreted this as RNF43 also being present at the inside of the nuclear rim [5, 7, 21]. However, the ER is tightly associated with the nuclear membrane in a continuous fashion [23], meaning that an ER-staining pattern can be easily mistaken for a location on the inside of the nuclear membrane. Second, cellular fractionation of overexpressed RNF43 has also been used to support a nuclear location [5, 7, 21], but given the tight association between the ER and the nuclear membrane, it is technically challenging to prevent ER contamination in nuclear fractions [23]. In fact, the fractionation experiments reported by Loregger et al. show that the Calnexin-positive ER fraction is completely isolated along with the nuclear fraction [5], thus not allowing a proper conclusion about nuclear vs ER location. Proteomic analyses dedicated to the nuclear envelope of three tissues identified over 5200 unique proteins, however, RNF43 was not observed (Dr. Eric Schirmer, personal communication) [24], further making a nuclear location less likely. Third, overexpression studies and staining of endogenous RNF43 provide strongly conflicting staining patterns. The HPA008079 and 8D6 antibodies yield a more or less homogenous nuclear staining pattern, while overexpressed RNF43 is negative inside the nucleus and shows at most staining of the nuclear border. Finally, epitope tagging endogenous RNF43 mostly fails to show its endogenous location. Tsukiyama et al. used a C-terminal HA-epitope in HEK293 cells, but failed to obtain a specific signal using IF [7]. Likewise, we were unable to detect 3xFLAG-tagged RNF43 in Caco-2 cells and can only identify a small number of 3xFLAG-positive OE19 cells. These latter cells show a cytoplasmic staining and no obvious signal in the nucleus, but given the low number of positive cells in only one cell line, we cannot draw a reliable generalized conclusion about RNF43’s intracellular location. Nevertheless, the difficulty to obtain signals with sensitive tags is in stark contrast to the readily obtained nuclear staining patterns observed with two commonly used RNF43 antibodies. As we show here that these antibodies are non-specific, we feel that currently no good evidence exists to support a nuclear location of RNF43.

Our results do not exclude that RNF43 indirectly affects nuclear functions. RNF43 was also shown to regulate non-canonical Wnt signaling by removing ROR1/2 from the membrane, and in concerted action with RSPO2/3 to affect BMPR1A receptor levels and BMP signaling [25–27]. Thus, besides regulating nuclear Wnt/β-catenin signaling, RNF43 can possibly also affect nuclear functions through these alternative routes, while not having to be located in the nucleus itself.

RNF43 antibodies have also been used to evaluate protein levels in tumor samples. Various conclusions were raised, such as RNF43 being overexpressed in liver cancers [8], to correlate with gastric and clear cell renal cancer patient survival [11–13], or to be lost in a subset of gastric and colorectal cancers [9, 10]. Jo et al. showed data in which RNF43 staining appeared to correlate with mutation status, as tumors with N-terminal truncating RNF43 mutations showed a negative staining [10]. However, in a more recent report, Omori et al. failed to find a good correlation between *RNF43* mutation status in pancreatic lesions and IHC using HPA008079 [14], which can be readily explained by the lack of antibody specificity that we observe. Based on our results, these and other reports in which RNF43 antibodies were used, should be interpreted cautiously, at least for the RNF43 protein aspects described in these papers.

Taken together, our results show that four tested RNF43 antibodies are not reliable tools to evaluate the intracellular location of RNF43 by IF and IHC, and also cannot detect endogenous RNF43 using immunoblotting. It also calls for a re-evaluation of the proposed nuclear functions of RNF43; are they the result of a direct effect of RNF43 protein within the nucleus, or an indirect consequence of RNF43 affecting one or more signaling routes? More RNF43 antibodies are commercially available. However, given the difficulty that we observe to detect endogenous RNF43, we feel that they are unlikely to detect RNF43 correctly. None of them have undergone the rigorous testing that we have performed here using cell lines in which the epitope is entirely lacking. Until such an analysis is done, we feel that they should not be used for research purposes, as this will potentially lead to misleading conclusions. If indeed no good antibodies are available and tagging endogenous RNF43 does not provide clear answers, it also means that we currently do not know the exact intracellular location of this important regulatory protein, an issue that can hopefully be resolved in the future.

## Materials and methods

### Cell lines and culture

OE19 and KM12 cells were maintained in RPMI-1640 culture medium containing 10% fetal bovine serum (FBS) and 1 mM sodium pyruvate. Caco-2, HEK293T, HCT116, DLD-1 and HT-29 cells were cultured in Dulbecco’s Modified Eagle’s medium (DMEM) supplemented with 10% FBS. Culture medium was changed every 2–3 days. All the cell lines were cultured in a humidified incubator maintained at 37°C with 5% CO2. All cell lines tested negative for mycoplasma based on the real-time PCR method at Eurofins GATC-Biotech (Konstanz, Germany). Identity of all cell lines and clones thereof, was confirmed by the Erasmus Molecular Diagnostics Department, using Powerplex-16 STR genotyping (Promega, Leiden, The Netherlands). RNF43 mutation status depicted in S1 Table was confirmed in all cell lines by Sanger sequencing and was consistent with those reported at COSMIC, the Catalog Of Somatic Mutations In Cancer (http://cancer.sanger.ac.uk).

### The generation of a RNF43 exon8-9 deletion DLD-1 cell line and RNF43-KO KM12 cell line

RNF43 exon8-9 deletion DLD-1 cells were generated via CRISPR-Cas9 genome editing. Two single guide RNAs (sgRNA) which target the introns between either exons 7 and 8 or exons 9 and 10 were designed using the following CRISPR design tool (http://crispor.tefor.net/), and cloned into pSpCas9 (BB)-2A-GFP (pX458), a gift from Feng Zhang (Addgene plasmid # 48138), using standard procedures [28]. The sgRNA sequences are listed in S2 Table. Cells were seeded into six-well plates and transfected with 600 ng of both pX458 plasmids, using Lipofectamine 2000 Transfection Reagent (ThermoFisher) following the manufacturer’s instructions. After transfection for 48 h, single GFP-positive cells were sorted out and plated into 96-well culture plates by a fluorescence activated cell sorter (FACS) FACSAria II cell sorter (BD Biosciences, San Jose, CA, USA). Three weeks later, genomic DNA was extracted from expanded single cell clones by QuickExtract DNA Extraction Solution (Epicenter, Madison, WI, USA). To confirm that the correct exon8-9 deletion was obtained, clones were first screened with primers flanking the deletion (S3 Table). Next, complete absence of exons 8 and 9 was confirmed with exon-specific primers (S3 Table). The generation of RNF43-KO KM12 cells has been described previously [17].

### Quantitative real-time PCR (qRT-PCR) and cDNA PCR

Briefly, total RNA was isolated using the NucleoSpin RNA II kit (Macherey-Nagel), after which the RNA was reverse transcribed with Primescript RT reagent kit (TaKaRa) according to the manufacturer’s instruction. Quantitative PCR was performed in the StepOne Real-Time PCR System (Applied Biosystems). Analyses were performed by using the StepOne version 2.0 software (Applied Biosystems) with the comparative ΔΔCT method and normalized with the human housekeeping gene GAPDH. All experiments were performed in triplicate. Primer sequences are provided in S4 Table.

To confirm the complete absence of exons 8 and 9 in the cDNA of DLD-1 ΔEX8-9 cells, a PCR was performed with flanking primers (S4 Table). Generated PCR products were run on gel and sequence-verified.

### Generation of cell lines with 3xFLAG-tag knock-in

Using CRISPR-Cas9 genome editing, we generated Caco-2 and OE19 cells with a knockin of the 3xFLAG-tag preceded by a SGGGSGGGS linker at the C-terminus of RNF43. To this aim, a sgRNA targeting before the stop codon of RNF43 exon10 was designed using the following CRISPR design tool (http://crispor.tefor.net/), and cloned into pSpCas9(BB)-2A-Puro (pX459) (S2 Table). A homology-directed repair (HDR) plasmid was acquired by cloning a PCR-generated 513bp fragment encompassing the stop codon of RNF43 into pEGFP-C1 (this plasmid was merely used as a backbone plasmid, not to express GFP). Next, the linker-3xFLAG sequence was cloned in frame with RNF43 using standard procedures. We noticed that Caco-2 cells harbor an uncommon heterozygous ACACCAT- ACATCAT variation in the 3’UTR. To avoid preferential targeting of the common allele, we also generated a HDR-plasmid carrying the ACATCAT variant using Q5 Site-directed Mutagenesis (New England Biolabs).

Caco-2 and OE19 cells were seeded in 10cm culture dishes and transfected with 3μg of pX459 and 12μg of HDR plasmid (6μg of each Caco-2 variant) using Lipofectamine 2000 Transfection Reagent. After transfection for 20h, cells were trypsinized and separated in the ratio of 1/7, 2/7 and 4/7. Cells of each ratio were equally seeded in three 10cm culture dishes. The next day, transfected cells were selected with 4μg/ml Puromycin for 3 days. Three weeks later, single clones were picked and seeded in a 96-well plate. Following Quickextract DNA isolation, correctly targeted clones were identified by PCR using primers in S5 Table. Subsequently, genomic DNA and cDNA of potential correct clones was verified using Sanger sequencing.

### Immunoblotting analysis

Immunoblotting was carried out using standard methods. Cells were lysed in 2× Laemmli sample buffer with 0.1 M dithiothreitol (DTT) and heated for 10 min at 95°C. Proteins were separated on 10% sodium dodecyl sulfate-polyacrylamide gel electrophoresis (SDS-PAGE). Proteins were then transferred to Immobilon-P PVDF membranes (Millipore, Bedford, MA,USA). Membranes were blocked 1 h with Odyssey blocking buffer (LI-COR-Biosciences, Lincoln, NE, USA) at room temperature and incubated overnight with primary antibodies at 4°C. After washing with TBS/0.05% Tween20 (TBST) buffer 10 min three times, the membranes were incubated for 1h with IRDye 680 goat anti-mouse (1:10.000, LI 926–68070), IRDye 800 goat anti-rabbit (1:5.000, LI 926–32211), or IRDye 680RD goat anti-rat (1:10.000, LI 926–68076) secondary antibodies (LI-COR-Biosciences), and then washed with TBST 10 min three times. The membranes were then scanned on the Odyssey Infrared Imaging System (LI-COR-Biosciences). Primary antibodies used were mouse anti-FLAG (1:1000, cat.# F1804, Sigma-Aldrich, St. Louis, MO, USA), rabbit anti-RNF43 HPA008079 (1:500, Lot 000005723, SigmaAldrich), rabbit anti-RNF43 ab84125 (1:500, Lot GR3296254, Abcam), rabbit anti-RNF43 ab217787 (1:1000, Lot 1015173–8, Abcam), and rat anti-RNF43 clone 8D6 (1:1000, a kind gift from Dr. Markus Gerhard, Technische Universität München, Munich, Germany). Mouse anti-β-actin (1:1000, cat.# sc-47778, Santa Cruz, CA, USA), and rabbit polyclonal to alpha Tubulin (1:2000, cat.# ab4074, Abcam) were used as loading controls.

For enhanced chemiluminescence (ECL)-based detection, Immobilon Block-CH (Chemiluminescent Blocker) blocking buffer was used (cat.# WBAVDCH01, Millipore). The primary antibodies were diluted in this blocking buffer. Membranes were washed with TBS containing 0.05% Tween 20 (TBST). The following secondary antibodies were used: Goat anti-mouse/HRP (1:10.000, cat.# A16078, ThermoFisher), goat anti-rabbit/HRP (1:10.000, cat.# P0448, DAKO), or rabbit anti-rat/HRP (1:10.000, cat.# P0450, DAKO). Membranes were then incubated with Immobilon ECL Ultra Western HRP Substrate (Millipore) and visualized by using Amersham Imager 600 (GE Healthcare).

### RNF43 immunofluorescence analysis

For RNF43 immunofluorescence we basically followed the procedure described by Neumeyer et al. [29]. In short, DLD-1, DLD-1 ΔEX8-9, and HCT116 cells were grown on cover slips in 12-well plates in DMEM/10%FCS without phenol red. In addition, we cultured HCT116 cells transfected with a C-terminal HA-tagged RNF43 expression plasmid (a kind gift from Dr. Markus Gerhard, Technische Universität München, Munich, Germany). Cells were washed with PBS, fixed in ice-cold methanol/acetone (1:1) for 15 min, followed by 3 washes in PBS. Next, cells were incubated at RT for 30 min with blocking/washing solution (3% bovine serum albumin (BSA), 1% saponin in PBS). Cells were then stained with either ab84125, ab217787, HPA-008079 or 8D6 antibody, all at 1:400 dilution, overnight at 4°C. RNF43-HA transfected HCT116 cells were co-stained with RNF43 antibodies and HA-Tag (6E2) Mouse mAb (#2367, Cell Signaling Technology) at a 1:400 dilution. After 3 washes with blocking/washing solution, the following secondary reagents were used: Donkey anti-Rabbit-Alexa 488 (cat.# A-21206), Goat anti-Rat-Alexa 594 (cat.# A-11007), Goat anti-Mouse-Alexa 488 (cat.# A-32723), Donkey anti-Mouse-Alexa 594 (cat.# A-21203); all from Thermo Fisher Scientific, at 1:400 dilution at RT for 1 h. Following 3 times washing with PBS containing 1% saponin, and one PBS wash, slides were mounted with Vectashield antifade mounting medium with DAPI (cat.# H-1200, Vector Laboratories, Burlingame, CA, USA). Images were captured by a Zeiss LSM510 Meta confocal laser scanning microscope using ZEN 2009 software with constant parameter setting.

### Using Tyramide SuperBoost to detect RNF43-3xFLAG-tag

RNF43-3xFLAG Caco2 cells, RNF43-3xFLAG OE19 cells, Caco2 cells and OE19 cells were grown on 8-well Lab-Tek II chamber sides (Thermo Scientific). After 48h cells were fixed in ice-cold methanol/acetone (1:1) for 15 min, followed by 3 washes in PBS and stained using Tyramide SuperBoost Kits (Alexa Fluor 488 Tyramide SuperBoost Kit, goat anti-mouse IgG, ThermoFisher) according to the manufacturers’ protocol. The optional step to block endogenous peroxidases was applied. Cells were stained with anti-FLAG antibody (1:500, cat.# F1804, Sigma-Aldrich) overnight at 4°C. Slides mounting and generation of images were performed as described above.

### Preparation of paraffin blocks from cell lines

The generation of formalin-fixed paraffin-embedded cell lines was basically performed as described previously [30]. In short, cells were grown in three 150cm^2^ plates until near-confluency. Next, the cells were scraped, washed with PBS, fixed in PBS-buffered 10% formalin and mixed with 1% agarose, prior to embedment in paraffin according to routine protocols. HCT116 cells transiently transfected with a FLAG-tagged RNF43 expression plasmid were also included.

### Immunohistochemistry

Immunohistochemical analyses for the RNF43 antibodies (all 1:200 diluted, except for ab217787 which was diluted 1:300) were performed in an automated stainer (Benchmark-Ultra, Ventana Medical Systems, Tucson, AZ). Sections were deparaffinized and pretreated with standard cell conditioning 1 solution (CC1) at 100°C for 64 min, followed by incubation with the specified antibodies at 37◦C for 60 min. The antibodies were visualized with the OptiView IHC DAB Detection Kit.

## Supporting information

S1 Fig Original immunoblot images of RNF43 antibodies. Pages 1–4 show the original blots corresponding to Fig 2. Page 5 shows an 8D6 immunoblot developed with the ECL-based secondary detection system not shown in Fig 2. Page 6 shows a FLAG-tag immunoblot to indicate the RNF43-FLAG protein transiently transfected in HEK293T cells. Page 7 shows the original immunoblot corresponding to Fig 6.
(PDF)

S2 Fig Larger images of the immunofluorescence analysis of RNF43 antibodies on DLD-1, DLD-1 ΔEX8-9, and HCT116 cells, shown in Fig 2. At the bottom of page 2 a higher intensity image is shown for ab84125 antibody, to better reveal the intracellular structures recognized non-specifically. Page 3 shows negative-control stainings with the secondary anti-rat and anti-rabbit antibodies. Scale bar, 25um.
(PDF)

S1 Table Cell lines used in this study. RNF43 mutation status is depicted. homo., homozygous; het., heterozygous.
(DOCX)

S2 Table The sgRNA sequences used for CRISPR-Cas9 genome editing. (DOCX)

S3 Table Primers used to identify DLD-1 clones with exon 8–9 deletion. (DOCX)

S4 Table Primers for qRT-PCR and for confirming exon 8–9 deletion in cDNA. (DOCX)

S5 Table RNF43-3xFLAG-tag clones screening primers. (DOCX)

## References

[1] Bugter JM, Fenderico N, Maurice MM. Mutations and mechanisms of WNT pathway tumour suppressors in cancer. Nat Rev Cancer. 2021 Jan;21(1):5–21. doi: 10.1038/s41568-020-00307-z 33097916

[2] Hao HX, Jiang X, Cong F. Control of Wnt Receptor Turnover by R-spondin-ZNRF3/RNF43 Signaling Module and Its Dysregulation in Cancer. Cancers (Basel). 2016 Jun 8;8(6): doi: 10.3390/cancers8060054 27338477 PMC4931619

[3] Koo BK, Spit M, Jordens I, Low TY, Stange DE, van de Wetering M, et al. Tumour suppressor RNF43 is a stem-cell E3 ligase that induces endocytosis of Wnt receptors. Nature. 2012 Aug 30;488(7413):665–9. doi: 10.1038/nature11308 22895187

[4] Yu F, Yu C, Li F, Zuo Y, Wang Y, Yao L, et al. Wnt/beta-catenin signaling in cancers and targeted therapies. Signal Transduct Target Ther. 2021 Aug 30;6(1):307. 34456337 10.1038/s41392-021-00701-5 PMC8403677

[5] Loregger A, Grandl M, Mejias-Luque R, Allgauer M, Degenhart K, Haselmann V, et al. The E3 ligase RNF43 inhibits Wnt signaling downstream of mutated beta-catenin by sequestering TCF4 to the nuclear membrane. Science signaling. 2015 Sep 8;8(393):ra90. 26350900 10.1126/scisignal.aac6757

[6] Neumeyer V, Brutau-Abia A, Allgauer M, Pfarr N, Weichert W, Falkeis-Veits C, et al. Loss of RNF43 Function Contributes to Gastric Carcinogenesis by Impairing DNA Damage Response. Cell Mol Gastroenterol Hepatol. 2021 Nov 11;11(4):1071–94. doi: 10.1016/j.jcmgh.2020.11.005 33188943 PMC7898035

[7] Tsukiyama T, Zou J, Kim J, Ogamino S, Shino Y, Masuda T, et al. A phospho-switch controls RNF43-mediated degradation of Wnt receptors to suppress tumorigenesis. Nat Commun. 2020 Sep 15;11(1):4586. doi: 10.1038/s41467-020-18257-3 32934222 PMC7492264

[8] Xie H, Xing C, Cao G, Wei B, Xu X, Song P, et al. Association of RNF43 with cell cycle proteins involved in p53 pathway. Int J Clin Exp Pathol. 2015;8(11):14995–5000. 26823834 PMC4713620

[9] Bond CE, McKeone DM, Kalimutho M, Bettington ML, Pearson SA, Dumenil TD, et al. RNF43 and ZNRF3 are commonly altered in serrated pathway colorectal tumorigenesis. Oncotarget. 2016 Sep 20. doi: 10.18632/oncotarget.12130 27661107 PMC5342576

[10] Jo YS, Kim MS, Lee JH, Lee SH, An CH, Yoo NJ. Frequent frameshift mutations in 2 mononucleotide repeats of RNF43 gene and its regional heterogeneity in gastric and colorectal cancers. Hum Pathol. 2015 Nov;46(11):1640–6. doi: 10.1016/j.humpath.2015.07.004 26297255

[11] Niu L, Qin HZ, Xi HQ, Wei B, Xia SY, Chen L. RNF43 Inhibits Cancer Cell Proliferation and Could be a Potential Prognostic Factor for Human Gastric Carcinoma. Cell Physiol Biochem. 2015;36(5):1835–46. doi: 10.1159/000430154 26184844

[12] Holm B, Barsuhn S, Behrens HM, Kruger S, Rocken C. The tumor biological significance of RNF43 and LRP1B in gastric cancer is complex and context-dependent. Sci Rep. 2023 Feb 23;13(1):3191. doi: 10.1038/s41598-023-30294-8 36823311 PMC9950470

[13] Zhu D, Shi X, Tian Y, Li H, Tang B, Zhang Z, et al. Combining expression of RNF43 and infiltration level of CD163(+) tumor associated macrophage predicts prognosis of clear cell renal cell carcinoma. Cancer Med. 2022 Sep 12. doi: 10.1002/cam4.5229 36097369 PMC9972079

[14] Omori Y, Ono Y, Tanino M, Karasaki H, Yamaguchi H, Furukawa T, et al. Pathways of Progression From Intraductal Papillary Mucinous Neoplasm to Pancreatic Ductal Adenocarcinoma Based on Molecular Features. Gastroenterology. 2019 Feb;156(3):647–61 e2. doi: 10.1053/j.gastro.2018.10.029 30342036

[15] Aoki Y, Mizuma M, Hata T, Aoki T, Omori Y, Ono Y, et al. Intraductal papillary neoplasms of the bile duct consist of two distinct types specifically associated with clinicopathological features and molecular phenotypes. The Journal of pathology. 2020 May;251(1):38–48. doi: 10.1002/path.5398 32100878

[16] Sakihama K, Koga Y, Yamamoto T, Shimada Y, Yamada Y, Kawata J, et al. RNF43 as a predictor of malignant transformation of pancreatic mucinous cystic neoplasm. Virchows Arch. 2022 Jun;480(6):1189–99. doi: 10.1007/s00428-022-03277-9 35066614

[17] Li S, Lavrijsen M, Bakker A, Magierowski M, Magierowska K, Liu P, et al. Commonly observed RNF43 mutations retain functionality in attenuating Wnt/beta-catenin signaling and unlikely confer Wnt-dependency onto colorectal cancers. Oncogene. 2020 Apr;39(17):3458–72. 32103169 10.1038/s41388-020-1232-5

[18] Yu J, Yusoff PAM, Woutersen DTJ, Goh P, Harmston N, Smits R, et al. The Functional Landscape of Patient-Derived RNF43 Mutations Predicts Sensitivity to Wnt Inhibition. Cancer research. 2020 Dec 15;80(24):5619–32. doi: 10.1158/0008-5472.CAN-20-0957 33067269

[19] Bradbury A, Pluckthun A. Reproducibility: Standardize antibodies used in research. Nature. 2015 Feb 5;518(7537):27–9. doi: 10.1038/518027a 25652980

[20] Acharya P, Quinlan A, Neumeister V. The ABCs of finding a good antibody: How to find a good antibody, validate it, and publish meaningful data. F1000Res. 2017;6:851. doi: 10.12688/f1000research.11774.1 28713558 PMC5499787

[21] Sugiura T, Yamaguchi A, Miyamoto K. A cancer-associated RING finger protein, RNF43, is a ubiquitin ligase that interacts with a nuclear protein, HAP95. Exp Cell Res. 2008 Apr 15;314(7):1519–28. doi: 10.1016/j.yexcr.2008.01.013 18313049

[22] Tu J, Park S, Yu W, Zhang S, Wu L, Carmon K, et al. The most common RNF43 mutant G659Vfs*41 is fully functional in inhibiting Wnt signaling and unlikely to play a role in tumorigenesis. Sci Rep. 2019 Dec 6;9(1):18557. doi: 10.1038/s41598-019-54931-3 31811196 PMC6898356

[23] Korfali N, Florens L, Schirmer EC. Isolation, Proteomic Analysis, and Microscopy Confirmation of the Liver Nuclear Envelope Proteome. Methods in molecular biology (Clifton, NJ. 2016;1411:3–44. doi: 10.1007/978-1-4939-3530-7_1 27147032

[24] Korfali N, Wilkie GS, Swanson SK, Srsen V, de Las Heras J, Batrakou DG, et al. The nuclear envelope proteome differs notably between tissues. Nucleus. 2012 Nov-Dec;3(6):552–64. doi: 10.4161/nucl.22257 22990521 PMC3515538

[25] Tsukiyama T, Fukui A, Terai S, Fujioka Y, Shinada K, Takahashi H, et al. Molecular Role of RNF43 in Canonical and Noncanonical Wnt Signaling. Molecular and cellular biology. 2015 Jun 01;35(11):2007–23. doi: 10.1128/MCB.00159-15 25825523 PMC4420922

[26] Radaszkiewicz T, Noskova M, Gomoryova K, Vondalova Blanarova O, Radaszkiewicz KA, Pickova M, et al. RNF43 inhibits WNT5A-driven signaling and suppresses melanoma invasion and resistance to the targeted therapy. Elife. 2021 Oct 27;10. doi: 10.7554/eLife.65759 34702444 PMC8550759

[27] Lee H, Seidl C, Sun R, Glinka A, Niehrs C. R-spondins are BMP receptor antagonists in Xenopus early embryonic development. Nat Commun. 2020 Nov 4;11(1):5570. doi: 10.1038/s41467-020-19373-w 33149137 PMC7642414

[28] Ran FA, Hsu PD, Wright J, Agarwala V, Scott DA, Zhang F. Genome engineering using the CRISPR-Cas9 system. Nature protocols. 2013 Nov;8(11):2281–308. doi: 10.1038/nprot.2013.143 24157548 PMC3969860

[29] Neumeyer V, Grandl M, Dietl A, Brutau-Abia A, Allgauer M, Kalali B, et al. Loss of endogenous RNF43 function enhances proliferation and tumour growth of intestinal and gastric cells. Carcinogenesis. 2019 Jun 10;40(4):551–9. doi: 10.1093/carcin/bgy152 30380024

[30] Dubbink HJ, Hollink I, Avenca Valente C, Wang W, Liu P, Doukas M, et al. A novel tissue-based β-catenin gene and immunohistochemical analysis to exclude familial adenomatous polyposis among children with hepatoblastoma tumors. Pediatr Blood Cancer. 2018 Jun;65(6):e26991. 29446530 10.1002/pbc.26991

