# Supplementary material for: Issues with RNF43 antibodies to reliably detect intracellular location
Source: PLoS One. 2023 Apr 6;18(4):e0283894. doi: 10.1371/journal.pone.0283894 (PMC10079101; doi:10.1371/journal.pone.0283894)

Supplemental Figure 1, page 1

original immunoblot images
HPA008079 Rabbit polyclonal antibody (fluorescence)

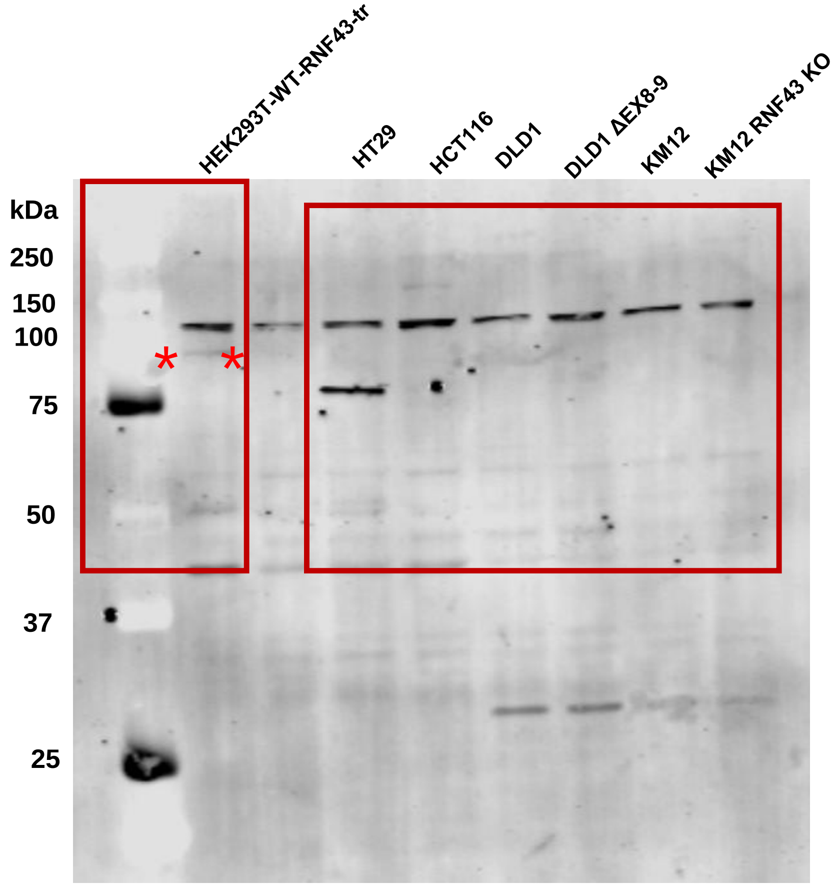

Actin (fluorescence)

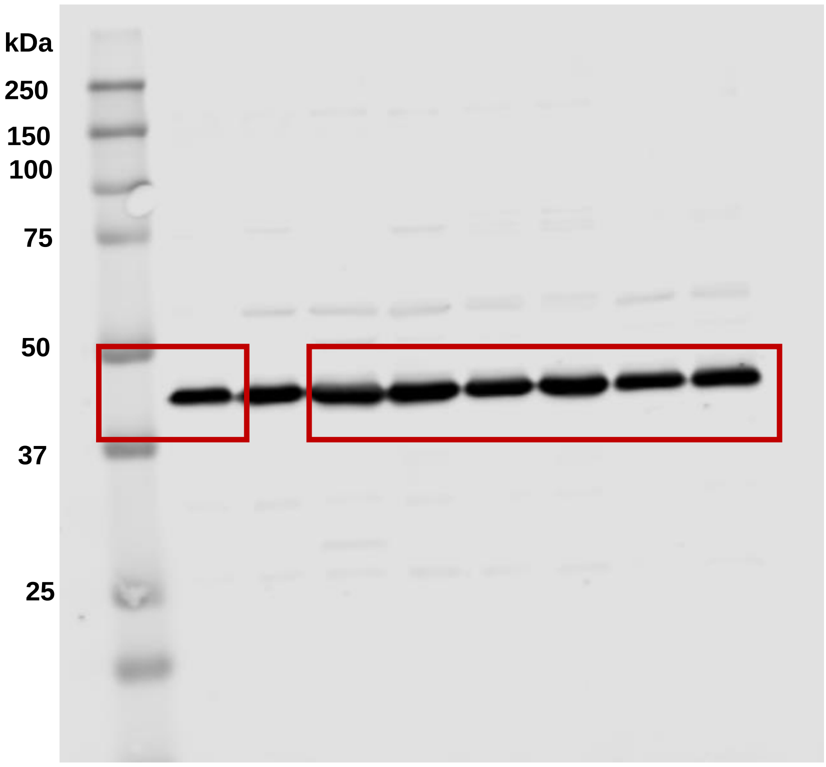

Supplemental Figure 1, page 2

original immunoblot images
8D6 Rat monoclonal antibody (fluorescence)

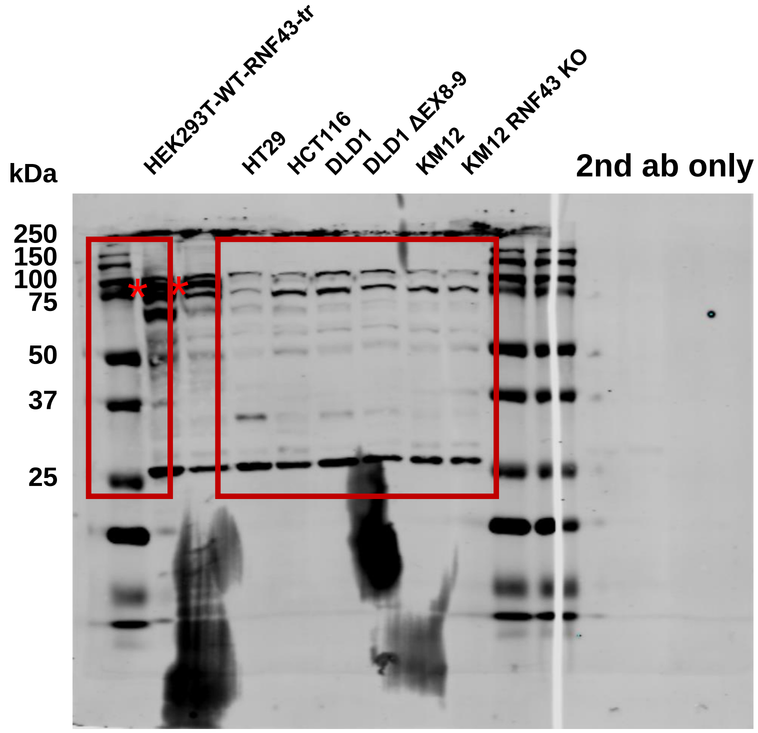

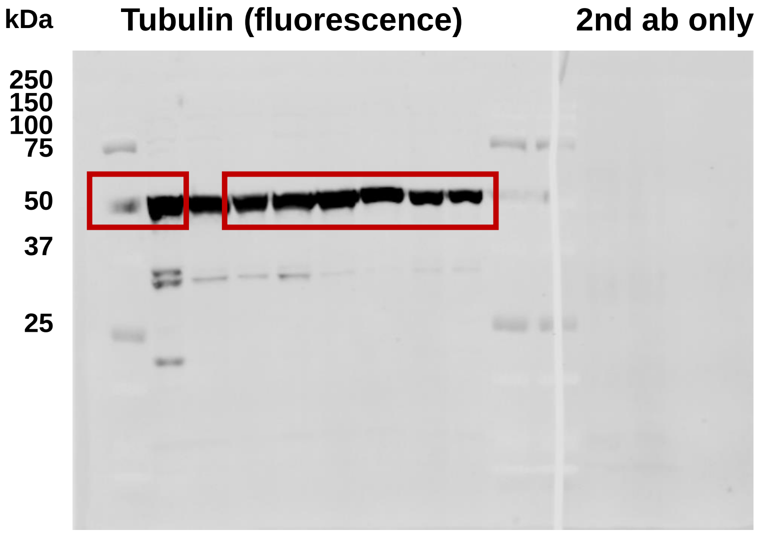

Supplemental Figure 1, page 3

original immunoblot images
Abcam ab84125 Rabbit polyclonal antibody (fluorescence)

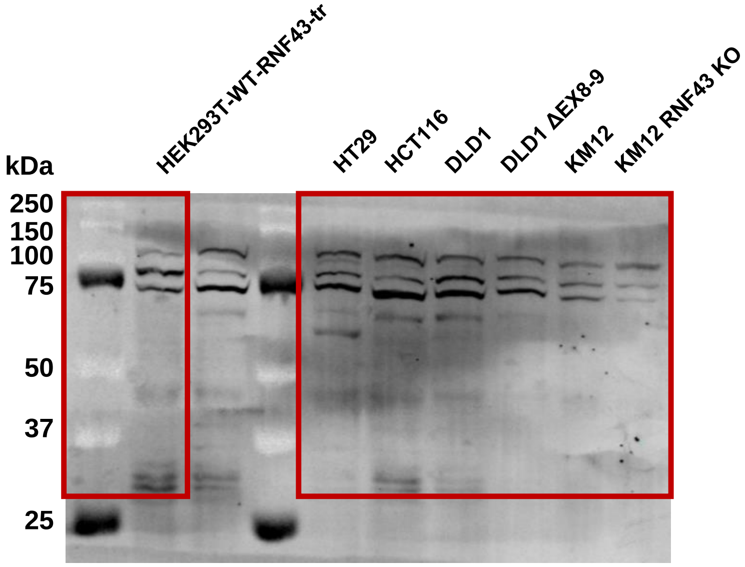

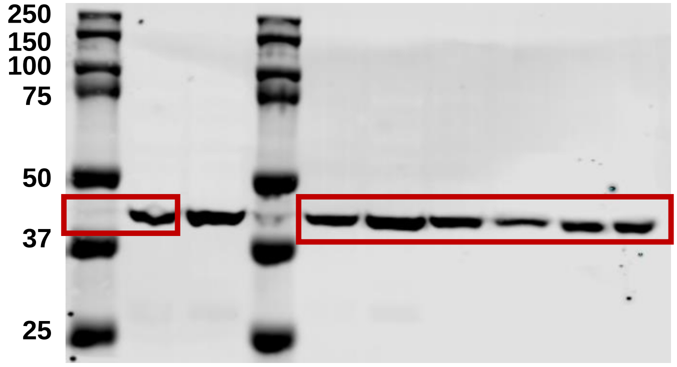

Supplemental Figure 1, page 4

original immunoblot images
Abcam ab217787 Rabbit polyclonal antibody (fluorescence)

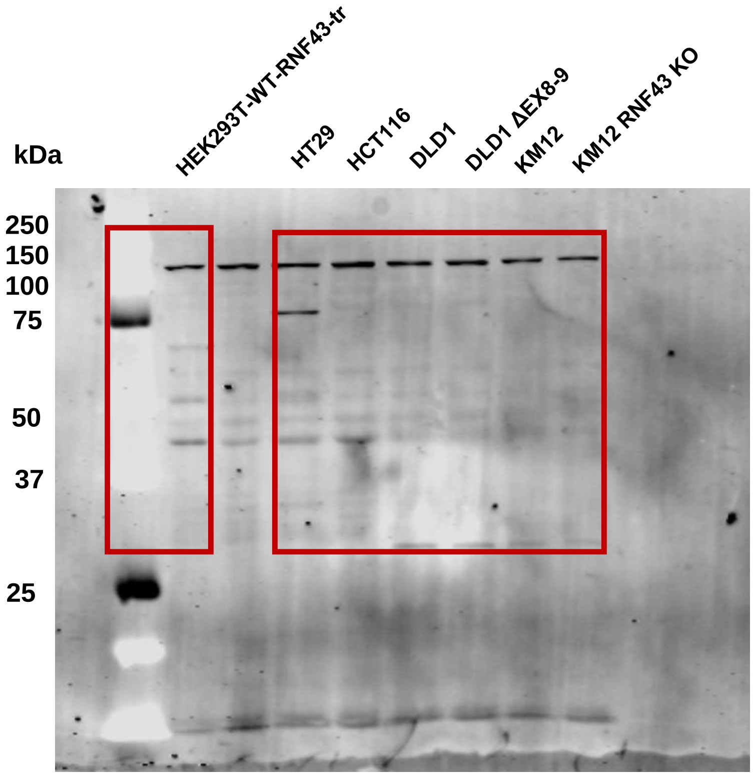

Actin (fluorescence)

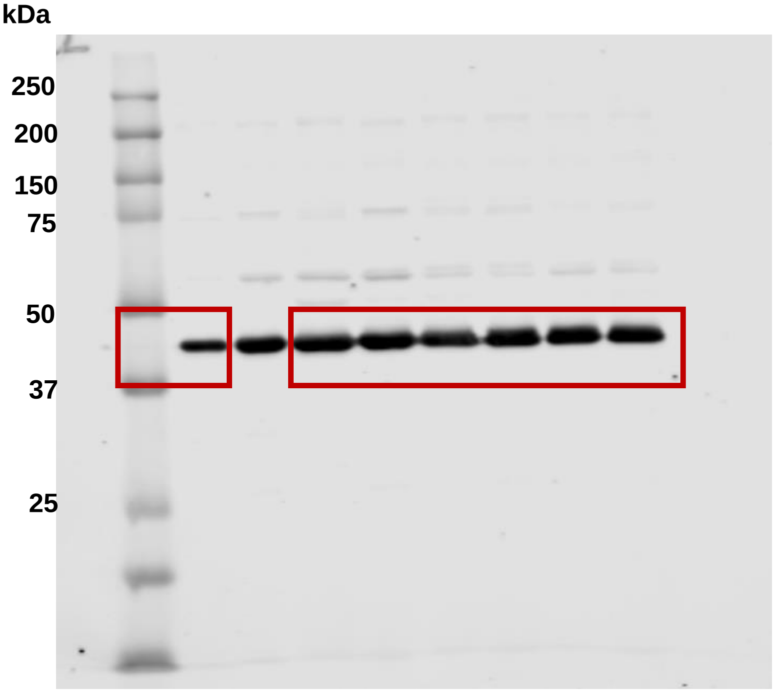

Supplemental Figure 1, page 5

Original immunoblot image
8D6 Rat monoclonal antibody (ECL; not shown in Figure 1)

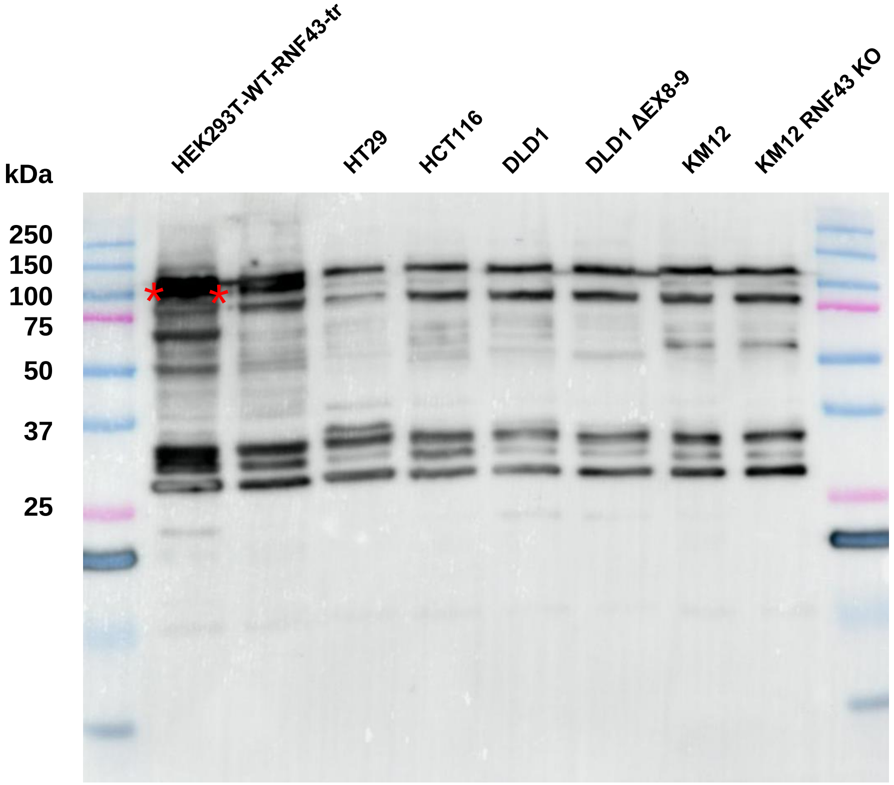

Supplemental Figure 1, page 6

original immunoblot images
Monoclonal ANTI-FLAG M2 antibody

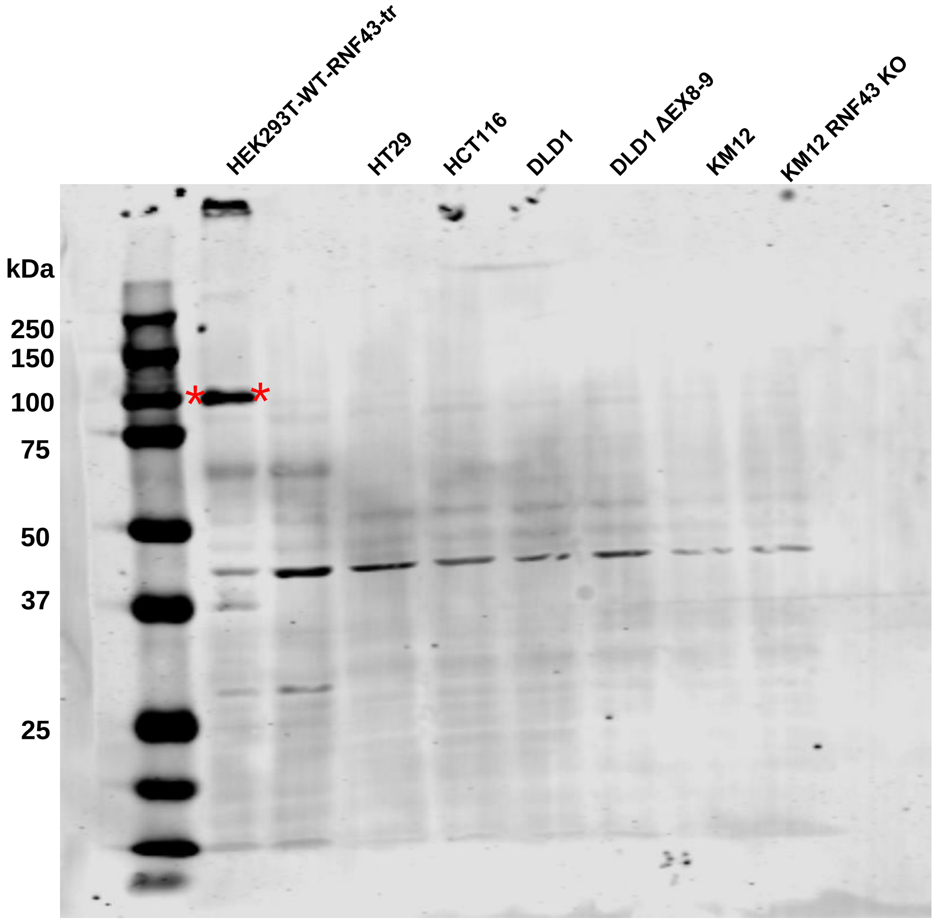

Supplemental Figure 1, page 7

Original IB images for Figure 4
Monoclonal ANTI-FLAG M2 antibody

RNF43-3xFLAG ANTI-FLAG (ECL)

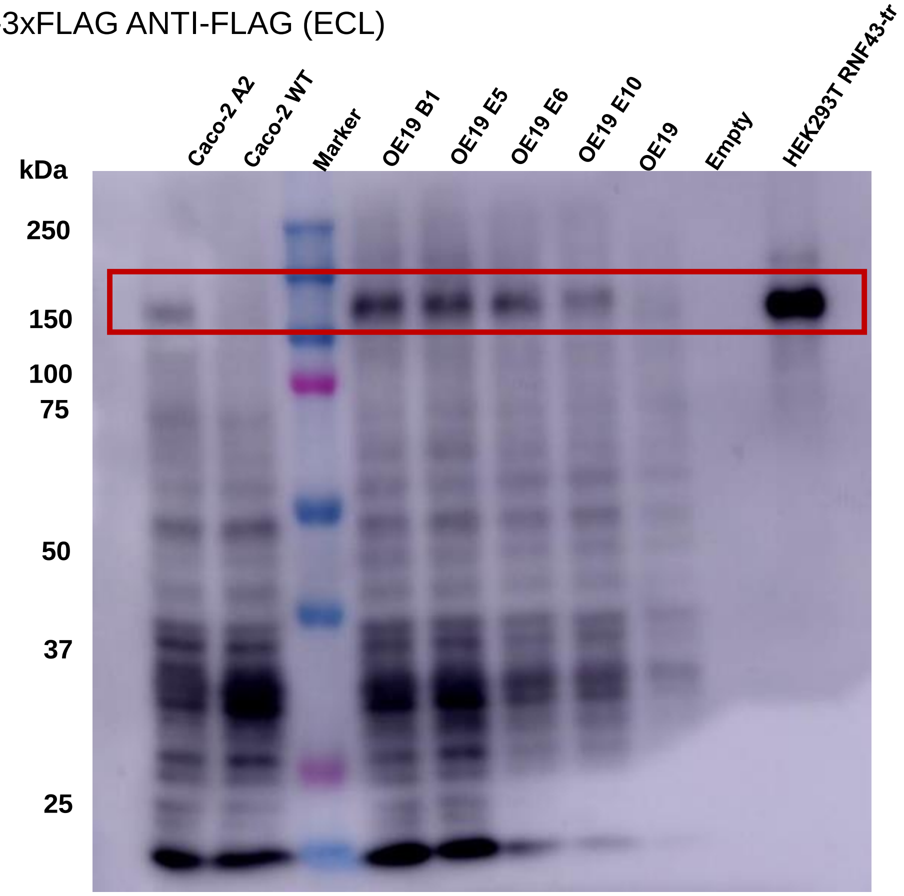

Actin (fluorescence)

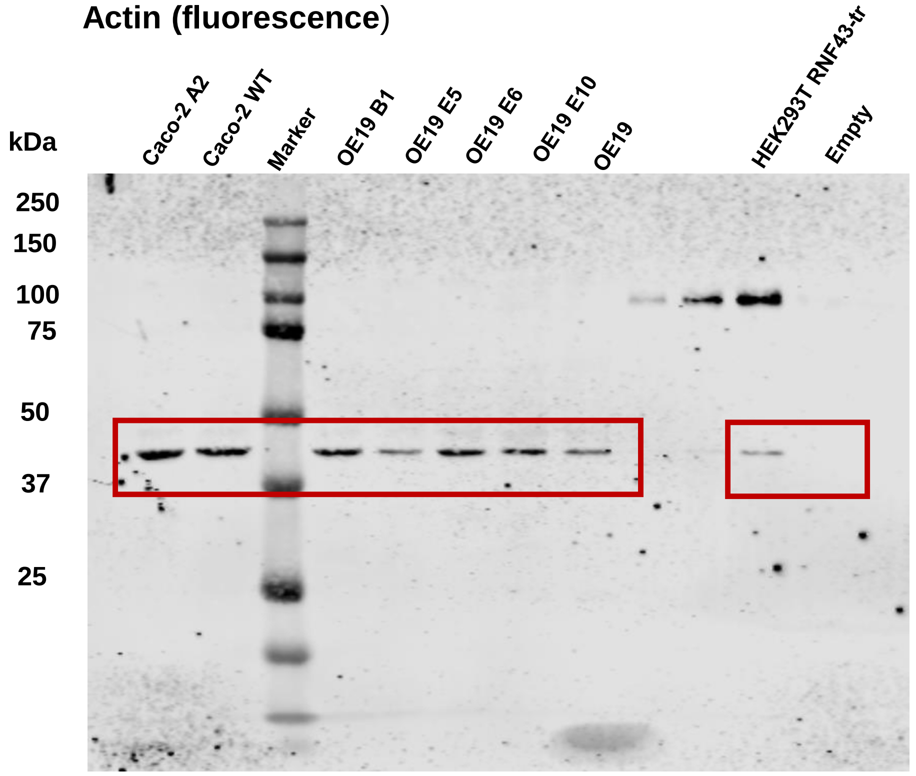

Supplement: S1 Fig — Pages 1–4 show the original blots corresponding to Fig 2. Page 5 shows an 8D6 immunoblot developed with the ECL-based secondary detection system not shown in Fig 2. Page 6 shows a FLAG-tag immunoblot to indicate the RNF43-FLAG protein transiently transfected in HEK293T cells. Page 7 shows the original immunoblot corresponding to Fig 6. (PDF) [file pone.0283894.s001.pdf]
