# Supplementary figures and images for: Issues with RNF43 antibodies to reliably detect intracellular location
Source: PLoS One. 2023 Apr 6;18(4):e0283894. doi: 10.1371/journal.pone.0283894 (PMC10079101; doi:10.1371/journal.pone.0283894)

## Supplemental Figure 2, page 1

**A**

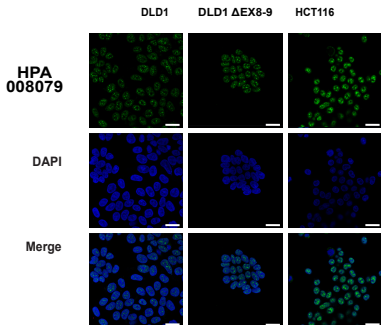

**B**

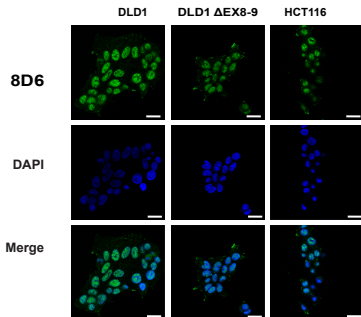

## Supplemental Figure 2, page 2

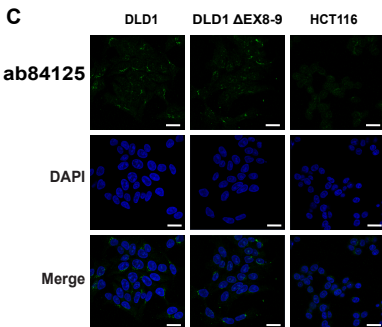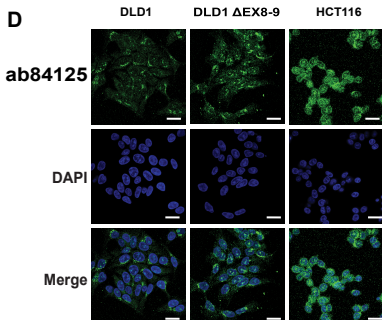

## Supplemental Figure 2, page 3

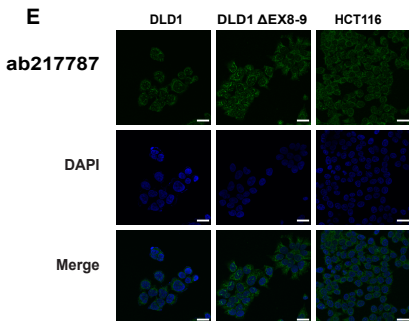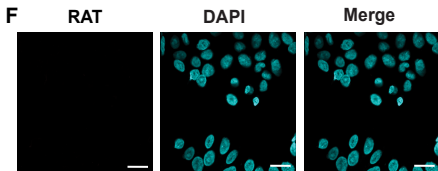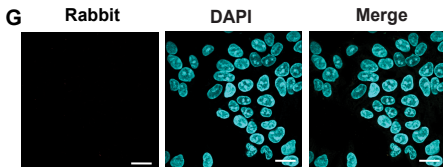

Supplement: S2 Fig — At the bottom of page 2 a higher intensity image is shown for ab84125 antibody, to better reveal the intracellular structures recognized non-specifically. Page 3 shows negative-control stainings with the secondary anti-rat and anti-rabbit antibodies. Scale bar, 25um. (PDF) [file pone.0283894.s002.pdf]
