# Supplementary material for: Issues with RNF43 antibodies to reliably detect intracellular location
Source: PLoS One. 2023 Apr 6;18(4):e0283894. doi: 10.1371/journal.pone.0283894 (PMC10079101; doi:10.1371/journal.pone.0283894)
Supplement: S1 Table — RNF43 mutation status is depicted. homo., homozygous; het., heterozygous. (DOCX) [file pone.0283894.s003.docx]

**S1 Table.** Cell lines used in this study. RNF43 mutation status is depicted. homo., homozygous; het., heterozygous.

| **Cell line name** | **RNF43 mutation status** |
| --- | --- |
| HCT116 | p.R117fs*41 homo. |
| DLD-1* | p.G659fs*41 het.+ p.L214M het.+ p.P231L het |
| KM12 | p.G659fs*41 homo. |
| HEK293T |  |
| HT-29 |  |
| Caco-2 |  |
| OE19 |  |

* Sequence analysis of cloned cDNA products from DLD-1 showed that the p.G659fs*41 and p.P231L are present on the same allele, while the p.L214M variant is present on the other chromosome. The P231L variant was shown to represent a common germline variant [5], while the L214M variant behaves like wild-type RNF43 in a β-catenin reporter analysis.
