# Supplementary material for: Issues with RNF43 antibodies to reliably detect intracellular location
Source: PLoS One. 2023 Apr 6;18(4):e0283894. doi: 10.1371/journal.pone.0283894 (PMC10079101; doi:10.1371/journal.pone.0283894)
Supplement: S2 Table — (DOCX) [file pone.0283894.s004.docx]

**S2 Table.** The sgRNA sequences used for CRISPR-Cas9 genome editing

| **sgRNA used for CRISPR-Cas9 genome editing** | **Forward primer(5'-3')** | **Reverse primer(5'-3')** |
| --- | --- | --- |
| RNF43 intron7 | caccgACCATTCCTATTCTACTGTG | aaacCACAGTAGAATAGGAATGGTc |
| RNF43 intron9 | caccGGTGCTACCCCTTTAGTTGG | aaacCCAACTAAAGGGGTAGCACC |
| RNF43 exon10 | caccgACTCGAGGAGCTGTGTGAAC | aaacGTTCACACAGCTCCTCGAGTc |
