# Supplementary material for: Issues with RNF43 antibodies to reliably detect intracellular location
Source: PLoS One. 2023 Apr 6;18(4):e0283894. doi: 10.1371/journal.pone.0283894 (PMC10079101; doi:10.1371/journal.pone.0283894)
Supplement: S3 Table — (DOCX) [file pone.0283894.s005.docx]

**S3 Table.** Primers used to identify DLD-1 clones with exon 8-9 deletion.

| **RNF43 deletion check** | **Forward primer(5'-3')** | **Reverse primer(5'-3')** |
| --- | --- | --- |
| Exon8-9 primers flanking deletion | CCTGTGTGTGCCATCTGTCT | ACTGAGCTGTGAGCATTGGT |
| Exon8 specific primers | GTCCTGATTCCTGGCAATTC | ATGGTGGCAGTTCTGCTTTC |
| Exon9 specific primers | CATGGCTCTTCCAGTGACTC | CTTCCCTCTGAAAACTCACC |
