# Supplementary material for: Issues with RNF43 antibodies to reliably detect intracellular location
Source: PLoS One. 2023 Apr 6;18(4):e0283894. doi: 10.1371/journal.pone.0283894 (PMC10079101; doi:10.1371/journal.pone.0283894)
Supplement: S4 Table — (DOCX) [file pone.0283894.s006.docx]

**S4 Table.** Primers for qRT-PCR and for confirming exon 8-9 deletion in cDNA

| **RNF43 deletion check** | **Forward primer(5'-3')** | **Reverse primer(5'-3')** |
| --- | --- | --- |
| Exon6&7 | CTGTGTGTGCCATCTGTCTG | GTCCGATGCTGATGTAACCA |
| Exon8&9 | CCTTCTGAATGGAGTTCTGAC | GCTAGGCCTGAACATCTCACA |
| Exon8-9 primers flanking deletion | TCCGCTTCAGCAGAGAACAG | TGGCTGGACATGGATTTGCT |
