# Supplementary material for: Issues with RNF43 antibodies to reliably detect intracellular location
Source: PLoS One. 2023 Apr 6;18(4):e0283894. doi: 10.1371/journal.pone.0283894 (PMC10079101; doi:10.1371/journal.pone.0283894)
Supplement: S5 Table — (DOCX) [file pone.0283894.s007.docx]

**S5 Table.** RNF43-3xFLAG-tag clones screening primers

| **primers** | **sequences** |
| --- | --- |
| RNF43-5intch-F | TCCAAAAAGGTTACCAGGTCCC |
| RNF43-5intch-R | GGTTTGGGTCCCCTCGTAT |
| RNF43-3intch-F | CACGGGTGGTATGGACGAACT |
| RNF43-3intch-R | CCAGGGTTGCCCCTGATGTA |
| RNF43-Gslink-F | AGCAAGCTGTGTCCGGAGGT |
| RNF43-FLAGlink-R | CCTGAACATCTCACTTGTCATCGT |
| RNF43-ex9_cbFin | ACCCACAGAGGAAAAGGCG |
